# Transcriptional analysis of human peripheral blood mononuclear cells stimulated by *Mycobacterium tuberculosis* antigen

**DOI:** 10.3389/fcimb.2023.1255905

**Published:** 2023-09-25

**Authors:** Jing Wei, Fangzheng Guo, Yamin Song, Kun Xu, Feiyang Lin, Kangsheng Li, Baiqing Li, Zhongqing Qian, Xiaojing Wang, Hongtao Wang, Tao Xu

**Affiliations:** ^1^ Laboratory Medicine Experimental Center, Laboratory Medicine College, Bengbu Medical College, Bengbu, China; ^2^ Anhui Province Key Laboratory of Immunology in Chronic Diseases, Bengbu Medical College, Bengbu, China; ^3^ Department of Immunology, Laboratory Medicine College, Bengbu Medical College, Bengbu, China; ^4^ Anhui Province Key Laboratory of Clinical and Preclinical Research in Respiratory Disease, Bengbu Medical College, Bengbu, China; ^5^ Department of Clinical Laboratory and Diagnostics, Laboratory Medicine College, Bengbu Medical College, Bengbu, China

**Keywords:** *Mycobacterium tuberculosis*, peripheral blood mononuclear cells, RNA sequencing, signaling pathway, *Mycobacterium tuberculosis* antigen (Mtb-Ag)

## Abstract

**Background:**

*Mycobacterium tuberculosis* antigen (Mtb-Ag) is a polypeptide component with a molecular weight of 10-14 kDa that is obtained from the supernatant of the H37Ra strain after heat treatment. It stimulates the activation and proliferation of γδT cells in the blood to produce an immune response against tuberculosis. Mtb-Ag is therefore crucial for classifying and detecting the central genes and key pathways involved in TB initiation and progression.

**Methods:**

In this study, we performed high-throughput RNA sequencing of peripheral blood mononuclear cells (PBMC) from Mtb-Ag-stimulated and control samples to identify differentially expressed genes and used them for gene ontology (GO) and a Kyoto Encyclopedia of Genomes (KEGG) enrichment analysis. Meanwhile, we used PPI protein interaction network and Cytoscape analysis to identify key genes and qRT-PCR to verify differential gene expression. Single-gene enrichment analysis (GSEA) was used further to elucidate the potential biological functions of key genes. Analysis of immune cell infiltration and correlation of key genes with immune cells after Mtb-Ag-stimulated using R language.

**Results:**

We identified 597 differentially expressed genes in Mtb-Ag stimulated PBMCs. KEGG and GSEA enrichment analyzed the cellular pathways related to immune function, and DEGs were found to be primarily involved in the TNF signaling pathway, the IL-17 signaling pathway, the JAK-STAT signaling pathway, cytokine-cytokine receptor interactions, and the NF-κB signaling pathway. Wayne analysis using GSEA, KEGG, and the protein-protein interaction (PPI) network showed that 34 genes, including PTGS2, IL-1β, IL-6, TNF and IFN-γ et al., were co-expressed in the five pathways and all were up-regulated by Mtb-Ag stimulation. Twenty-four DEGs were identified using qRT-PCR, including fourteen up-regulated genes (SERPINB7, IL20, IFNG, CSF2, PTGS2, TNF-α, IL36G, IL6, IL10, IL1A, CXCL1, CXCL8, IL4, and CXCL3) and ten down-regulated genes (RTN1, CSF1R CD14, C5AR1, CXCL16, PLXNB2, OLIG1, EEPD1, ENG, and CCR1). These findings were consistent with the RNA-Seq results.

**Conclusion:**

The transcriptomic features associated with Mtb-Ag provide the scientific basis for exploring the intracellular immune mechanisms against Mtb. However, more studies on these DEGs in pathways associated with Mtb-Ag stimulation are needed to elucidate the underlying pathologic mechanisms of Mtb-Ag during Mtb infection.

## 1 Introduction

Tuberculosis (TB) is a deadly infectious disease that is caused by *Mycobacterium tuberculosis* (Mtb). There were 10.6 million new TB cases and 1.6 million deaths in 2021, according to the World Health Organization (Bagcchi, 2023). It is estimated that at least 2 billion people (25%-30% of the world’s population) are carriers of latent tuberculosis (LTBI) (Seeberg, 2023). Although Bacillus Calmette-Guerin (BCG) has protected tens of millions of people from TB infections over the past century, BCG’s shortcomings, such as its poor effectiveness in the prevention of TB in adults and its varying effectiveness across different regions and populations, have made it harder to control TB at its source.

Although combination chemotherapy with anti-tuberculosis drugs has becoming increasingly sophisticated, the emergence of multi-drug-resistant and extensively drug-resistant strains and co-infections with HIV have led to an increased year-over-year death rate. The interactions between Mtb and the host immune system are very complex, and our understanding of the pathogenesis and protective immune response during TB infection remains incomplete (Wang et al., 2019). Mtb-Ag is derived from Mtb H37Ra, a peptide antigen that is released from the cytoplasm of Mtb after being autoclaved at 121°C for 20 min (Boom et al., 1994). Mtb-Ag activates γδ T cells, promotes their proliferation *in vitro*, and helps diagnose TB and LTBI. An important part of immune surveillance, peripheral blood mononuclear cells (PBMC) contain all of the key immune cells necessary for anti-Mtb activity, reflecting the body’s innate resistance (Li et al., 2022). Many studies have shown that the mRNA transcription levels of specific genes in PBMC can be used as a marker of diseases such as active tuberculosis and enterocolitis syndrome (Wu et al., 2020). However, the intracellular immune mechanism of Mtb-Ag has not been fully reported. Transcriptome sequencing is an emerging technology for transcriptome analysis using new-generation sequencing technology, which can quickly transcript certain tissues or cells after intervention and systematically evaluate changes in gene expression levels. With advantages that include high resolution, high throughput, high sensitivity, and convenient use, transcriptome sequencing has been widely used in medical, animal, plant, and other research fields (Stark et al., 2019). It is particularly suitable for identifying immune response dynamics and gene regulatory networks. This study used transcriptome sequencing technology to study the changes in PBMC gene expression after Mtb-Ag exposure and to explore the genes associated with Mtb-Ag infection. This would permit a better understanding of the changes in various cytokines that occur following Mtb-Ag stimulation and the impact of these changes on regulating multiple immune pathways. Our findings highlight the indispensable role of the innate immune pathway in the body’s fight against Mtb infection (Zhang Y et al., 2021), thereby providing a theoretical basis for an in-depth analysis of the inherent immune mechanisms against Mtb-Ag.

## 2 Materials and methods

### 2.1 Ethics statement and participant inclusion criteria

Eight peripheral blood samples (4 males and 4 females) were collected from healthy adults, aged 21 ± 2 years, from students of Bengbu Medical College, all of whom were in normal health, had physical examination with normal chest X-ray, and had no history of infectious diseases such as tuberculosis. Written informed consent was obtained for each subject and approved by the Human Ethics Committee of Bengbu Medical College (2022-68).

### 2.2 Culture of Mtb H37Ra and preparation of Mtb-Ag

Mtb-Ag was prepared according to the previous reports (Boom et al., 1994; Peng et al., 2008). Briefly, Mtb H37Ra was inoculated into 7H11 agar medium at 37°C and incubated for 21 days. Reference, strain H37Ra was inoculated into 7H11 agar medium at 37°C and incubated for 21 days. Then it was cultured in Sauton’s medium for 4 to 6 weeks to the late log phase, and the mycobacterial cells were harvested, 4 500 rpm, and centrifuged for 30 min. Washed three times with saline and once with ultra-pure water, then re-suspended in two volumes of ultra-pure water to cell pellets, then heating at 121°C for 20 min. Soluble Mtb-Ag was collected from a supernatant of heat-treated Mtb cells, and concentrated to 1 mg/ml. 4°C storage to be used. Mtb-Ag after extraction in this study was identified by gel and mainly used to identify its function and specificity by detecting the proliferation ratio of γδ T cells in human peripheral blood mononuclear cells by flow cytometry technique. Briefly, Mtb-Ag (5 μg/mL) was used to stimulate PBMCs (1×10^6^/mL). The cells were supplemented with rhIL-2 (50 U/mL) every 3 days, cells were collected on day 9 and proliferation of human peripheral blood γδ T cells was detected by flow cytometry (only IL-2 as control group).

### 2.3 PBMC isolation and Mtb-Ag stimulation

Peripheral blood was collected from 8 individuals, divided into two groups, no treatment as a control group and Mtb-Ag stimulation group; total 16 samples, 8 samples in the control, and 8 samples in the stimulation group. A single replicate is used for each donor in the control and stimulation groups. A total of 20 ml of peripheral blood was collected from each donor and placed in a vacutainer containing EDTA-K2 as an anticoagulant. Blood samples were processed within 2 hours of isolation. Following the manufacturer’s instructions, PBMCs were isolated via gradient centrifugation using a lymphocyte isolation medium (TBD, China). Briefly, blood was first diluted 1:1 with RPMI 1 640 (Gibco, USA), carefully layered on lymphoprep™ solution in a conical tube, and centrifuged at 2 000 rpm for 20 minutes. The PBMC layer was then collected in another conical tube, which was washed twice with RPMI 1640 and centrifuged at 1 500 rpm for 10 min. Cells were resuspended in RPMI 1640 medium containing 5% FBS (Gibco), 5% autologous serum, and 1% triple antibody (Penicillin-streptomycin-gentamicin mixed solution) (Solarbio). The cell concentration was adjusted to 1×10^6^/mL and placed at 1 mL/well into 24-well culture plates. Approximately 10 × 10^6^ PBMCs per donor were collected and either incubated with Mtb-Ag (5 µg/mL) or untreated for 6 h in a 5% CO_2_ incubator at 37°C. All procedures were performed in a BSL-2 laminar flow hood under sterile conditions to prevent endotoxin contamination, and all samples were processed under the same conditions.

### 2.4 RNA sample extraction and quality inspection

Total RNA was isolated from treated PBMCs using TRIzol (Invitrogen, CA, USA) and chloroform according to the provided instructions. RNA integrity, including RNA integrity number (RIN) values and 28s/18s ratios, was detected using Agilent 2100, and RNA concentration was measured using a Nanodrop UV analyzer. RNA was measured based on OD260/280 and OD260/230 to predict nucleic acid purity. A 1% agarose gel was prepared for electrophoresis, and a gel imaging system was used for imaging. Samples were sent to Shanghai Personalbio Technology Co., Ltd. (Shanghai, China) for RNA-sequencing and bioinformatics analyses.

### 2.5 RNA sequencing and bioinformatics analysis

Using the NovaSeq sequencing platform, the raw data (Rawdata) obtained from sequencing was filtered using Cutadapt software to obtain clean sequences (clean reads). The clean reads were compared with reference genome sequences using HISAT2 software, and expression was normalized using FPKM.

To understand the function of DEGs, we performed GO and KEGG enrichment analyses to identify relevant signaling pathways. The GO pathway includes three parts: molecular function (MF), cellular component (CC), and biological process (BP). KEGG has powerful graphical features as an integrated chemical, genomic, and systemic functional information database. We identified differentially expressed genes at different levels of each KEGG Pathway (https://www.genome.jp/kegg/pathway.html) and identified the metabolic and signaling pathways in which the differentially expressed genes are mainly involved, thereby permitting a comprehensive understanding of the disease. Results were visualized using the Sangerbox platform (http://sangerbox.com/), and GO enrichment and KEGG Pathway analyses were performed using the GSEA website. Gene sets with NES ≥ 1.0, nominal P value < 0.05, and FDRq value ≤0.25 were identified as meaningful.

PPI networks in meaningful signaling pathways were obtained using STRING’s online analysis website (https://string-db.org/) and exported in TSV format. The obtained source file was imported into the open-source software platform Cytoscape (https://cytoscape.org/) for visual analysis. The “MCODE” plugin of Cytoscape was utilized with default parameters to identify the most important clustering module in the PPI network. Another plugin, “Cytohubba”, was used to assign values to each gene using a topological network algorithm. The top 10 genes with the highest maximal clique centrality (MCC) values based on centrality, eccentricity, and radiality were selected as the hub genes in the PPI network.

### 2.6 Validation of quantitative real-time PCR

Gene expression was assessed using qRT-PCR. EasyScript^®^ One-Step gDNA Removal and cDNA Synthesis SuperMix (Transgen, Beijing, China) were used to reverse transcription cDNA. Each RT-PCR reaction consisted of a 20 µL system of 1 µg RNA, 1 µL Anchored Oligo (dT)_18_ primer (0.5 µg/µl), 10 µL 2 × ES reaction mix, 1 µl EasyScript^®^ RT/RI enzyme mix, 1 µl gDNA remover, and RNase-free water, which were amplified to generate cDNA in a T100™ Thermal Cycler (BIO-RAD, USA) for 15 min at 42°C, followed by 5 s at 85°C. The resultant mixture was then diluted 5-fold and stored at -80°C. qRT-PCR analyses were performed with the LightCycler^®^ 96 real-time PCR System (Roche Diagnostics GmbH, Mannheim, Germany), with each 20 µl sample containing 2 µl cDNA, 10 µl 2 × *PerfectStart*™ Green qPCR SuperMix, 0.4 µl of each primer, and 7.2 µl of nuclease-free water. Analyses were performed with 96-well plates (Roche) with thermocycler settings of 94°C for 30 s, 45 cycles of 94°C for 5 s, 60°C for 30 s, and 72°C 10 s. Samples were analyzed in triplicate, and melt curve analyses were performed to examine amplified PCR product specificity. Sangon Biotech (Shanghai) Co. Ltd. synthesized primers using NCBI database mRNA sequences (**Supplementary Table 1**). Relative gene expression was normalized to β-actin and calculated via the 2^−△△Ct^ approach.

### 2.7 Statistical analysis

All bioinformatics analyses were performed using R software. Data were analyzed using GraphPad Prism (Version 9.0, GraphPad Software, CA, USA) and SPSS (Version 26, IBM, New York, USA). A two-tailed *P*-value < 0.05 was considered statistically significant. **** represents *P* < 0.0001, *** represents *P* < 0.001, ** represents *P* < 0.01, and * represents *P* < 0.05.

## 3 Results

### 3.1 Summary of RNA sequence data

**Figure 1:**
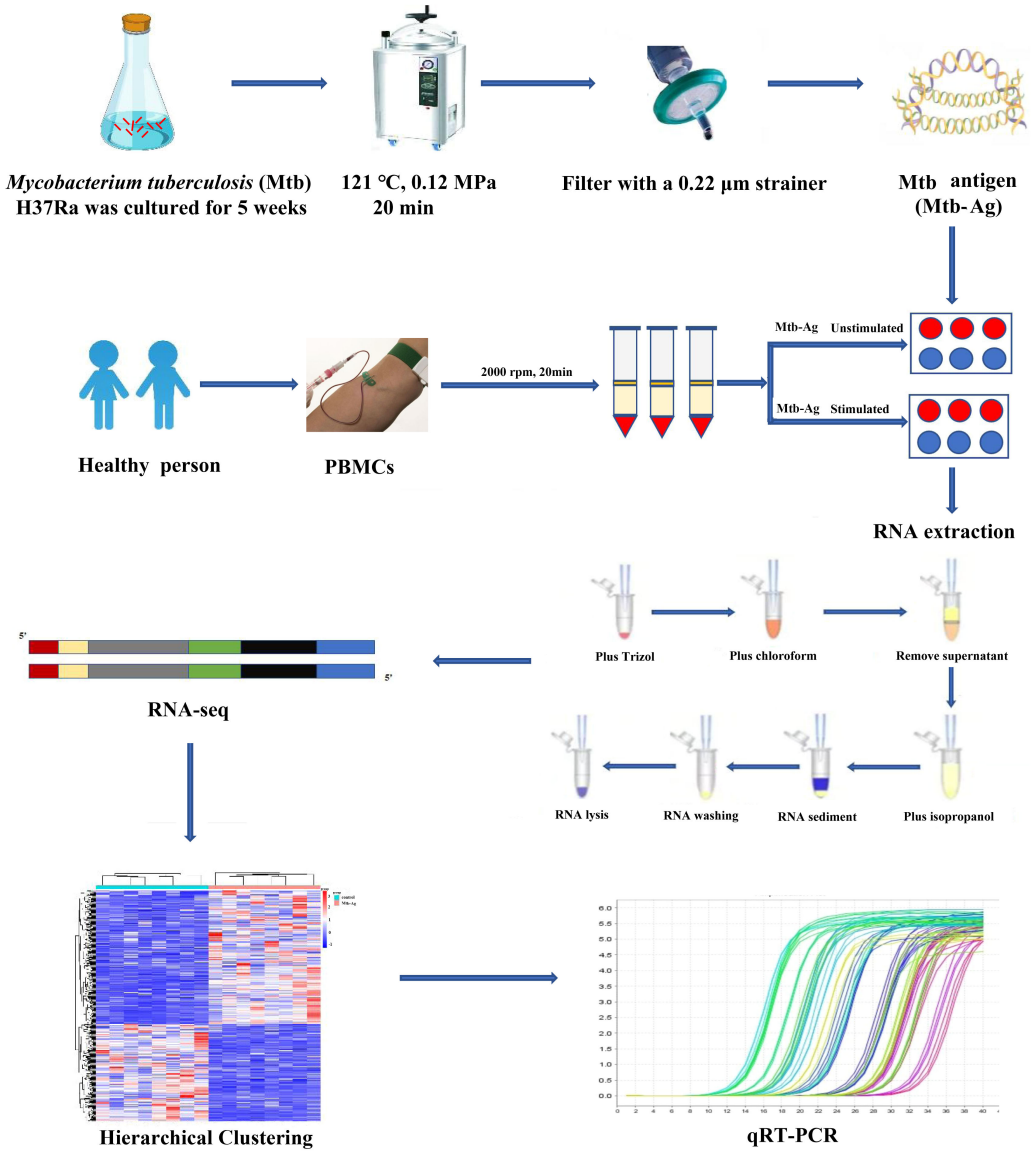
Transcriptome sequencing diagram.

The study flowchart is shown in **Figure 1**. The functionality and specificity of the extracted Mtb-Ag were characterized in **Supplementary Figure 1**. To explore the mechanism behind the pathogenic effects of Mtb-Ag on PMBCs, we performed RNA-seq analysis (**Supplementary Table 2**). The sequencing data of 16 samples (8 Mtb-Ag-stimulated samples and 8 controls) are shown in **Table 1**. 698.3 million RawReads were generated, and 655.26 million clean reads were left after removing low-quality reads and joint sequences. Between 96.07% and 96.86% of the readings in each sample were mapped to mapped reads, 96.73% to 97.13% were mapped to unique mapped reads, and 2.87% to 3.27% were mapped to multiple mapped reads. Q30 represents a sequencing error rate of 0.1%, and Q30 in this study is all above 93.73%, indicating that the sequencing error rate was minimal, the sequencing data quality was high, and the samples met the needs of this study.

**Table 1:** Sequencing transcriptome data statistics.

Sample	RawReads	CleanReads	Q30 (%)	Mapped Reads	Unique Mapped Reads	Multiple Mapped Reads
Con1	48721428	45486296	94.73	43932218 (96.58%)	42603398 (96.98%)	1328820 (3.02%)
Con2	43278528	40421500	94.42	38975823 (96.42%)	37776049 (96.92%)	1199774 (3.08%)
Con3	42602026	40099924	94.52	38664578 (96.42%)	37455494 (96.87%)	1209084 (3.13%)
Con4	37671790	35578636	94.39	34288697 (96.37%)	33243023 (96.95%)	1045674 (3.05%)
Con5	44712100	42098826	94.05	40546536 (96.31%)	39269695 (96.85%)	1276841 (3.15%)
Con6	46420570	43367876	94.11	41784164 (96.35%)	40472358 (96.86%)	1311806 (3.14%)
Con7	44449644	41599180	94.48	40292300 (96.86%)	39007034 (96.81%)	1285266 (3.19%)
Con8	44822452	42229428	94.14	40820087 (96.66%)	39647859 (97.13%)	1172228 (2.87%)
Ag1	44860346	42055296	94.48	40580226 (96.49%)	39371760 (97.02%)	1208466 (2.98%)
Ag2	47496828	44422158	94.5	42832166 (96.42%)	41516597 (96.93%)	1315569 (3.07%)
Ag3	42260300	39839074	94.65	38274656 (96.07%)	37108058 (96.95%)	1166598 (3.05%)
Ag4	43641472	40992358	94.55	39518294 (96.40%)	38328798 (96.99%)	1189496 (3.01%)
Ag5	40709060	38384040	94.36	37070561 (96.58%)	35947309 (96.97%)	1123252 (3.03%)
Ag6	38425822	36005352	93.73	34795069 (96.64%)	33753385 (97.01%)	1041684 (2.99%)
Ag7	42370264	39702098	94.56	38361032 (96.62%)	37106985 (96.73%)	1254047 (3.27%)
Ag8	45878036	42982442	94.4	41553552 (96.68%)	40346647 (97.10%)	1206905 (2.90%)

Ag and Con respectively correspond to the Mtb-Ag-stimulated and unstimulated groups, with 1-8 corresponding to eight parallel replicates.

### 3.2 Differentially expressed genes

**Figure 2:**
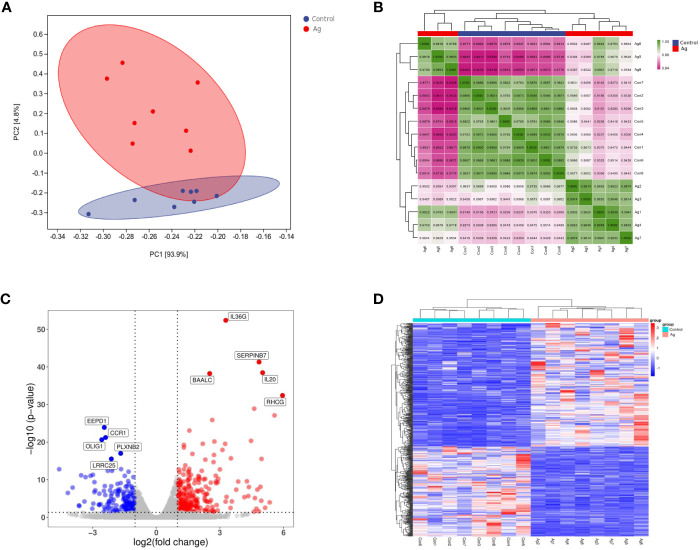
Differentially expressed genes (DEGs) identified in PBMCs stimulated with Mtb-Ag compared with control cells. **(A)** Principal component analysis of DEG transcripts. **(B)** Correlation heatmap of different samples. **(C)** A volcano plot highlighting DEGs between controls and Mtb-Ag-stimulated samples (log10p-value *vs*. log2FC). Blue and red dots with negative and positive change values correspond with down-regulated and up-regulated DEGs. **(D)** Hierarchical clustering of transcripts that were significantly (≥2-fold change) up-regulated (red) or downregulated (blue) in Mtb-Ag-stimulated PBMCs.

Principal component analysis and sample correlation tests were performed on 16 sequenced samples (8 Mtb-Ag-stimulated samples and 8 controls) at the transcriptome level. The correlation coefficients of 16 sequenced samples were all higher than 0.9, indicating obvious heterogeneity between the samples (**Figures 2A, B**). The Pearson correlation coefficient was used to represent gene expression level correlations between samples. The closer the correlation coefficient was to 1, the higher the similarity of expression patterns between samples. The above information indicates that the high throughput sequencing data of 16 samples was of high quality and met the requirements for subsequent transcriptome analysis.

Using DESeq2 software, 597 differentially expressed genes between Mtb-Ag stimulated and unstimulated groups were obtained, of which 345 were up-regulated and 252 were down-regulated (**Supplementary Table 3**). Stratified cluster analysis showed a difference in the expression pattern of DEGs between stimulated and unstimulated groups, and a volcano map was drawn to visualize the distribution of these DEGs. Significant differences (multiple expression differences | log_2_FoldChange | > 1, significant *P* values < 0.05) between the two groups of samples were marked with different colors. The genes with large differential expression multiples were distributed on both sides of the center line. In contrast, the genes with significant differential expression appeared higher in the figure (**Figure 2C**). These DEGs were visualized using a heat map (**Figure 2D**) and confirmed that Mtb-Ag stimulation was associated with a significant change in PBMC gene expression profile compared with control unstimulated samples.

### 3.3 GO function and KEGG pathway enrichment analysis of DEGs

**Figure 3:**
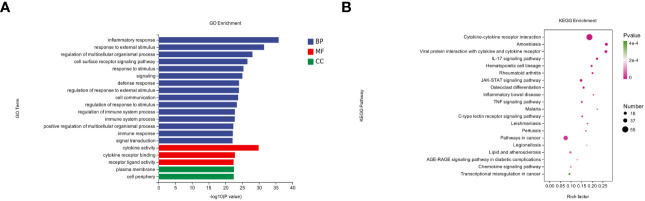
GO annotations and KEGG pathway analysis results for DEGs. **(A)** The top 20 biologic processes, cellular components, and molecular function GO terms are shown (*P* < 0.05; unique gene number of GO terms > 2). **(B)** The top 20 positively enriched KEGG pathways are shown in a bubble chart with enriched pathways on the y-axis and enrichment scores on the x-axis. A positive correlation between bubble size and the number of pathway-related genes was observed, with a larger pathway enrichment *P*-value associated with an increase in the degree of pink coloration of that bubble.

To further understand the pathologic effects of Mtb-Ag on PBMCs, we performed an enrichment analysis of the GO and KEGG pathways in DEGs. 8485 GO terms and 271 KEGG pathways were associated with these DEGs. GO analysis showed that following Mtb-Ag stimulation, the GO entries of PBMC differential genes were mainly enriched in the biological process (BP) category, followed by molecular function (MF) and cell component (CC) (**Figure 3A**). Further enrichment analysis was conducted on the GO entries of differential genes under BP, CC, and MF. **Figure 3A** shows that the biological processes with the most enriched GO entries were mainly involved in immune processes, particularly the inflammatory response, the response to external stimuli, and the regulation of multicellular organismal processes. The molecular function enrichment map shows that differential genes were mainly involved in the molecular functions of cytokine activity, cytokine receptor binding, and receptor-ligand activity. Classifying cell components with low GO concentrations mainly involved plasma membrane and cell periphery components. The pathways containing DEGs were annotated and enriched by KEGG, and 271 signaling pathways were screened. KEGG pathway annotation results suggest that these DEGs mainly involve human disease and tissue system-related pathways. Such pathways include cytokine-cytokine receptor interaction, the IL-17 signaling pathway, the JAK-STAT signaling pathway, the TNF signaling pathway, pathways in cancer, the NF-κB signaling pathway, and the C-type lectin receptor signaling pathway (**Figure 3B**).

### 3.4 Identification of hub DEGs

**Figure 4:**
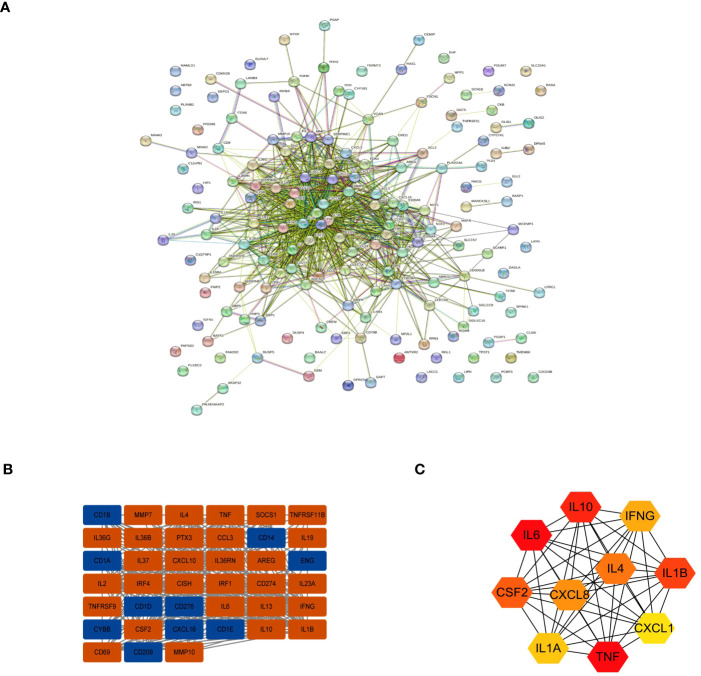
Interactions between the DEGs. **(A)** The PPI network. **(B)** The most significant module in the PPI network. **(C)** The Top 10 hub DEGs based on maximal clique centrality (MCC).

To further evaluate interactions between many DEGs and to screen out the key nodes that may play essential roles in the network, we imported the PPI network constructed with the STRING online website for all DEGs into Cytoscape software. We used the MCODE plugin to identify relevant sub-networks to identify DEGs that were the most critical clustering modules (**Figures 4A, B**). The top 10 pivotal DEGs were identified with “Cytohubba” with the MCC method and included IL10, IL6, CSF2, IL1A, TNF, CXCL1, IL1B, IFNG, IL4, and CXCL8 (**Figure 4C**).

### 3.5 Immune cell infiltration and correlation analysis of hub genes

**Figure 5:**
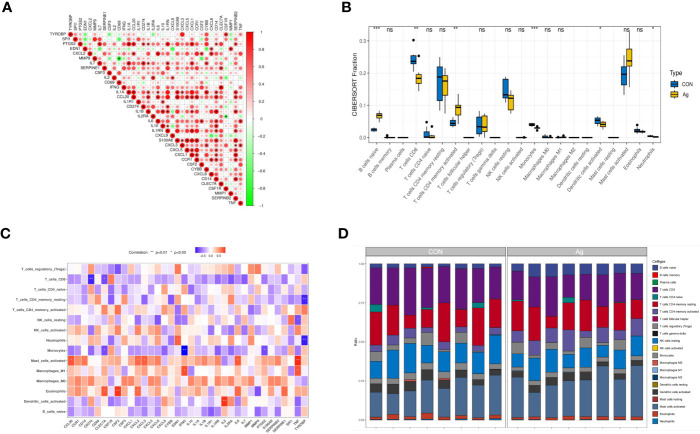
Interactions between the DEGs. **(A)** Correlation analysis of 36 DEGs. Red and green colors represent positive and negative correlations, respectively. **(B)** Boxplots of differences in immune infiltration between unstimulated and Mtb-Ag-stimulated PBMCs. **(C)** A heatmap showing correlations between 36 DEGs and infiltrating immune cells. **(D)** The relative abundance of 22 infiltrated immune cells between unstimulated and Mtb-Ag-stimulated PBMCs. (*P < 0.05, **P < 0.01, ***P < 0.001, ns, not significant.).

Further analyses revealed correlations between DEGs. CLEC7A had a strong synergistic effect with IFNG (coefficient = 0.96), and MMP9 had a significant antagonistic impact with CD69 (coefficient = - 0.86). Most of these genes were positively correlated (**Figure 5A**). To further confirm the role of immune cells using immune infiltration analysis, we found that six kinds of immune cells were significantly different in the Control group *vs*. the Mtb-Ag group: naïve B cells (*P* < 0.001), CD8 T cells (*P* < 0.01), CD4 memory activated T cells (*P* < 0.01), monocytes (*P <*0.001), activated dendritic cells (*P* < 0.05) and neutrophils (*P* < 0.05) (**Figure 5B**). Correlations between immune cell infiltration and DEGs are shown in **Figure 5C**. **Figure 5D** shows immune infiltration analysis based on the CIBERSORT algorithm, showing differences in the proportion of 22 infiltrating immune cell types between the two groups. This suggests that alterations in the function of specific immune cells in the immune microenvironment and differences in secreted cytokines after Mtb-Ag stimulation of PBMCs play an essential role in tuberculosis.

### 3.6 GSEA of all genes in Mtb-Ag-stimulated PBMCs

**Figure 6:**
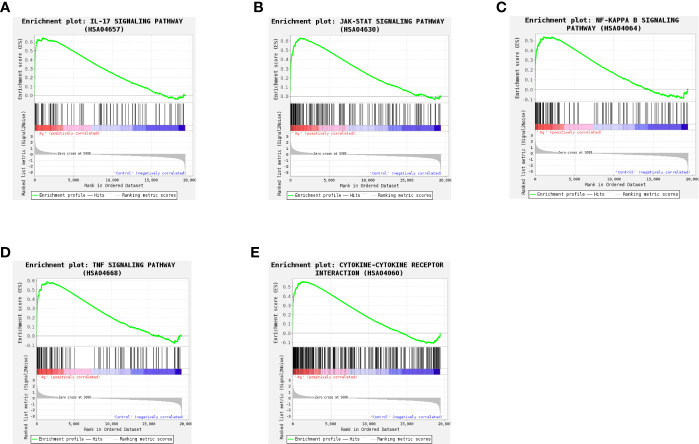
Gene set differences between Mtb-Ag-stimulated PBMCs and controls as illustrated using a gene set enrichment analysis (GSEA) approach. Enrichment plots for five GSEA pathways that were enriched in Mtb-Ag-stimulated PBMCs relative to controls. A gene set was considered significantly enriched if P <0.05. **(A)** IL-17 signaling pathway **(B)** JAK-STAT signaling pathway **(C)** NF-kB signaling pathway **(D)** TNF signaling pathway **(E)** Cytokine-Cytokine receptor interactions.

To more fully understand the whole-transcriptomic changes observed following Mtb-Ag stimulation, GSEA was performed. GSEA is focused on the whole gene set expression rather than DEGs. As shown in **Supplementary Table 4** and **Figure 6**, GSEA-based KEGG analysis indicated that the IL-17 signaling pathway (**Figure 6A**), the JAK-STAT signaling pathway (**Figure 6B**), the NF-κB signaling pathway (**Figure 6C**), the TNF signaling pathway (**Figure 6D**), and Cytokine-Cytokine receptor interactions (**Figure 6E**) were significantly enriched, and closely related to TB. Higher absolute times normalized enrichment fraction (NSE) values and smaller P- and FDR represented more significant enrichment. Through further analysis of these pathways, we found that the NF-κB signaling pathway is crucial for the induction of oxidative stress.

### 3.7 PPI network construction and functional analysis of the PBMC signaling pathways stimulated by Mtb-Ag

**Figure 7:**
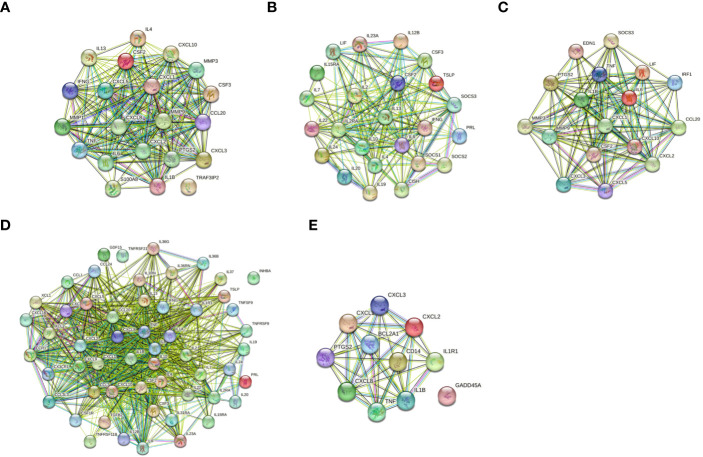
Construction of PPI networks. **(A)** The PPI network of the IL-17 signaling pathway. **(B)** The PPI network of the JAK-STAT signaling pathway. **(C)** The PPI network of the TNF signaling pathway. **(D)** The PPI network of cytokine-cytokine receptor interactions. **(E)** The PPI network of the NF-kappa B signaling pathway.

**Figure 8:**
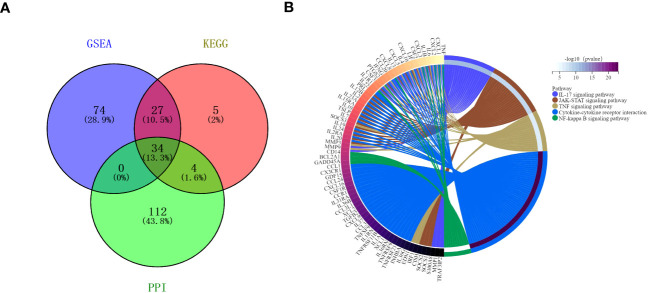
Interactions between the DEGs. **(A)** A Venn diagram illustrating the common overlapping genes in the GSEA, KEGG, and PPI analyses. These overlapping genes were related to five signaling pathways. Genes analyzed via the GSEA, KEGG, and PPI approaches are denoted in purple, pink, and green circles, respectively. In total, 34 genes overlapped across all three of these analyses. **(B)** Distribution of DEGs among the five signaling pathways.

The protein-protein interactions of 5 signaling pathways were obtained from the STRING database (**Figure 7**). Thirty-four related DEGs were found in five signaling pathways, all analyzed using Wayne analysis of three bioinformatic methods (GSEA, KEGG, and PPI network) (**Figure 8A**). PTGS2, CSF2, CXCL3, IL1B, CCL20, IL6, CXCL2, CXCL1, MMP9, SOCS3, TNF, IRF1, CSF3, IFNG, CXCL8, TRAF3IP2, IL20, IL24, IL2RA, IL19, IL7, IL10, IL15RA, IL36G, INHBA, TNFRSF21, IL1R1, TNFRSF9, IL1A, IL36RN, XCL1, BCL2A1, and GADD45A were the 34 DEGs that were shared between the three bioinformatic methods and were all up-regulated following Mtb-Ag exposure. The Sangerbox platform analyzed the visualization output of the five DEG pathways (**Figure 8B**).

### 3.8 RNA-seq validation

**Figure 9:**
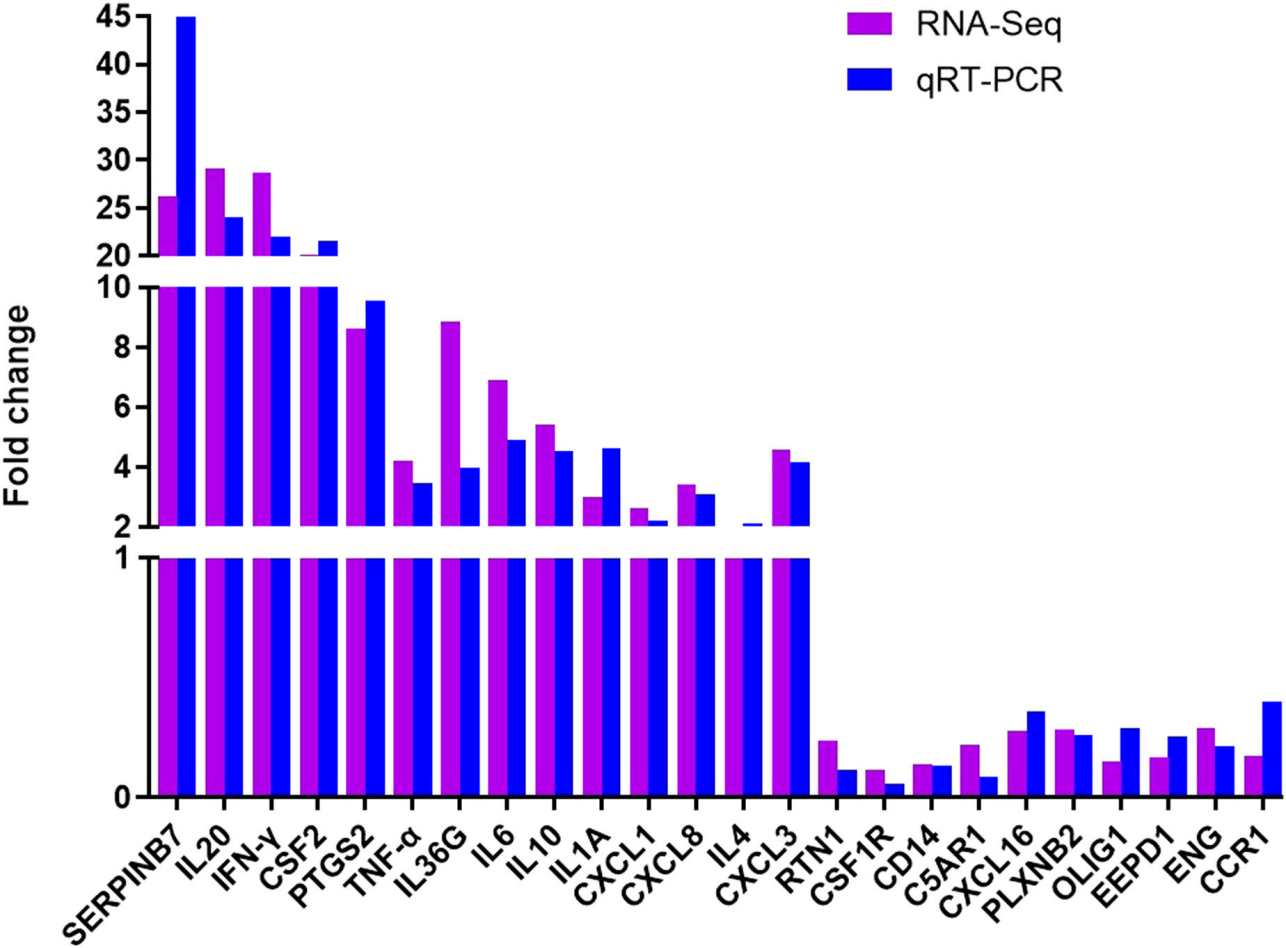
Validation of transcriptomic sequencing results using qRT-PCR.

To verify the accuracy of our RNA-seq analyses, we selected 14 up-regulated DEGs and 10 down-regulated DEGs for qRT-PCR-based validation. We found that the expression levels of these genes were comparable with what was observed in our RNA-seq analyses, thus confirming the accuracy and credibility of our transcriptomic results (**Figure 9**). (*P < 0.05, ** *P <*0.01, *** *P <*0.001, and **** *P <*0.0001)

## 4 Discussion

A major public health problem worldwide, TB is the most common fatal disease that is caused by the single infectious pathogen Mtb. There was a 3.6% increase in the incidence of TB in 2021 compared with 2020 (Chakaya et al., 2021; Bagcchi, 2023). The pathogenic mechanism of TB is complex. Improved diagnosis, prevention, and treatment strategies are urgently needed to control the spread of TB. In recent years, due to the gradual increase in multidrug-resistant TB, the co-occurrence of HIV with TB, and the coronavirus pneumonia (COVID-19) outbreak in 2020, TB’s global prevention and control has faced unprecedented challenges. Identifying and studying new Mtb antigens is critical to understanding TB’s pathogenesis and developing preventative vaccines.

Mtb-Ag is a peptide antigen with a molecular weight of 10-14 kDa released into the supernatant of the attenuated strain H37Ra after heat treatment. The immune mechanism between Mtb-Ag and infected host cells is not fully understood. This study identified intact mRNA with significant changes in Mtb-Ag stimulated PBMCs compared with unstimulated control PBMCs. Our sequencing results had high levels of quality and reliability. Bioinformatics analysis, GO terms enrichment, and KEGG enrichment identified DEG species and potential function. Identifying important biological functional changes can help us understand the molecular mechanisms of TB. A total of 597 DEGs, 345 up-regulated genes and 252 down-regulated genes, were found to differ between the Mtb-Ag stimulation group and the negative control group. GO enrichment analysis identified significant changes in DEGs that were primarily involved in the inflammatory response, the response to external stimuli, the regulation of multicellular organismal processes, and the cell surface receptor signaling pathway. KEGG enrichment results showed that DEGs stimulated by Mtb-Ag are mainly related to cytokine-cytokine receptor interactions, the JAK-STAT signaling pathway, the TNF signaling pathway, the IL-17 signaling pathway, the NF-κB signaling pathway, and the C-type lectin receptor signaling pathway. These pathways are closely related to tuberculosis’s pathogenesis (Turrin and Plata-Salamán, 2000; Rawlings et al., 2004; Wu et al., 2021; Zhang T et al., 2021). This suggests that GO and KEGG were practical ways to further investigate the mechanism of action of Mtb-Ag in tuberculosis.

The next stage of our research was to use fewer biomarkers to identify hub DEGs in Mtb-Ag, which could indicate subgroups of TB patients with specific clinical characteristics. Therefore, we predicted central DEGs in the PPI network and noted strong interactions between TNF, IL-6, IL-10, IL-1β, CSF-2, IL-4, CXCL8, IFN-γ, IL-1α, and CXCL1, all of which are strongly associated with tuberculosis. IFN-γ, TNF, and IL-1β, are essential cytokines for the immune control of Mtb (Mayer-Barber et al., 2011). TNF-α is an early inflammatory factor produced by Th1 cells, which participates in the humoral response (Sugiyama et al., 2023). TNF-α induces a local immune response and stimulates vascular endothelial cells, causing a large number of leukocytes to accumulate in the inflammation area and stimulating monocytes to secrete more inflammatory cytokines, such as IL-1, IL-6, and TNF, which enhance the Tc-mediated destruction of the infected cells (Alvarez-Sekely et al., 2023). It induces apoptosis of Mtb macrophages, which also mediate the growth inhibition of Mtb, leaving Mtb in a dormant state (Tan et al., 2023).TNF-α is a critical component of host innate immunity against pathogen challenges (Mootoo et al., 2009). The lack of TNF-α leads to the rapid progression of the inflammatory response and uncontrolled bacterial growth, while excess TNF-α has been shown to impair lung function (Kiran et al., 2016; Guler et al., 2021). IFN-γ is a pleiotropic cytokine mainly produced by activated CD4^+^ T cells and CD8^+^ T cells, γδ T cells, and natural killer cells (Kiflie et al., 2023). In tuberculosis infection, IFN-γ enhances the expression of MHCII-like molecules on the surface of macrophages, promotes macrophage presentation of antigens and its activation, and initiates the body’s immune response against infection, which is proved to significantly inhibit Mtb from surviving and reproducing in the body (Dhiman et al., 2023). IFN-γ can induce the differentiation of Th0 cells to Thl cells, and activate more effector cells and cytotoxic T cells (CTL), which are crucial for killing or inhibiting Mtb (Poladian et al., 2023). PTGS2 is one of the proximal genes regulated by lncRNA when Mtb, PTGS2, and IL-6 infect cells are up-regulated at the same time, and the ester substance tretinoin inhibits the expression of PTGS2 enhances the immunoreactivity of monocyte-derived macrophages (monocyte-derived macrophages, MDMs) and mediates the killing effect of MDMs on Mtb (Tamgue et al., 2021). PTGS2 also assists in synthesizing the prostaglandin PEG2, which can induce apoptosis of Mtb-infected macrophages (Liu et al., 2020). Combined with our transcriptomic sequencing results, we hypothesize that Mtb-Ag up-regulates PTGS2 by activating the IL-17, TNF, and NF-κB signaling pathways, making the immune response an important tool against tuberculosis. CD4^+^ T cells and IFN-γ cooperate and are one of the most important and perfect partners for anti-TB cellular immunity. However, in recent years it has been found that in the absence of IFN-γ, CD4^+^ T cells can still effectively drive the activation of bone marrow-derived macrophages (BMDMs) to control the progression of inflammation. It has previously been reported using a “co-culture” model that CD4^+^ T cells activate the transcription factor HIF-1α by secreting granulocyte-macrophage colony-stimulating factor (GM-CSF/CSF2), thereby establishing a link between GM-CSF and HIF-1α. The link between GM-CSF and HIF-1α was established, demonstrating the bactericidal activity of CSF2 in this “pathway” and assisting CD4^+^ T cells in suppressing Mtb infection (Van Dis et al., 2022). CSF2 promotes Tfh1 cell polarization by activating dendritic cells (DCs) in a CD40-dependent manner (Korniotis et al., 2022), and also up-regulates anti-apoptosis-related genes such as Bcl-2 and HSP27 to maintain the survival of macrophages that have been infected with Mtb. The ability of macrophages to secrete CSF2 is significantly negatively correlated with intracellular bacterial load. Reduced levels of GM-CSF were found in macrophages isolated from the peripheral blood of patients with active tuberculosis, and the killing capacity of the cells for Mtb was significantly reduced (Mishra et al., 2022). IL-1α and IL-1β gene variants may somewhat increase TB susceptibility (Salum et al., 2020). Producing the pro-inflammatory cytokines IL-1β and TNF-α helps inhibit Mtb infection in granulomas and prevent the spread of Mtb. Mtb-induced IL-1β acts in an autocrine manner in human macrophages and plays a key role in limiting the growth of Mtb in the cell through increased TNF signaling and the subsequent up-regulation of caspase-3 (Sarkar et al., 2015). IL-10 trends higher in TB patients (Hasan et al., 2012), but it is not a specific marker of TB. However, IL-10 may be involved in the mutual regulation of the levels of CSF-2 and CSF-3 to balance the levels of these three cytokines, which is associated with the risk of tuberculosis (Gao et al., 2015). IL-4 is an anti-inflammatory cytokine that down-regulates IFN-γ to affect TB patients adversely (Qi et al., 2014). However, partial single nucleotide polymorphisms of IL-4 have been found to reduce the risk of severe TB. IL-4 is mainly secreted by CD4^+^ T lymphocytes, which mediate Th2 type immune response, activate the differentiation of Th0 cells to Th2 type cells and inhibit the immune response of Th1 type cells, and induce the activation of aberrant macrophages to increase the susceptibility to Mtb (Pooran et al., 2019). IL-6 is secreted by TLR-2-expressing cells and regulates the production of pro- and anti-inflammatory cytokines, playing a more prominent role in the immune process against early Mtb infection (Kamimura et al., 2003). Its role is particularly critical when the TB bacterial load is high (Jang et al., 2004; Torrado and Cooper, 2010; He et al., 2018). IL-2 is a unique class of active cytokines produced by T-lymphocytes that limit the replication of Mtb by activating macrophages and stimulating the production of cytotoxic T-lymphocytes directed against Mtb antigens (Chesov et al., 2015; Shen et al., 2022).

The classical view is that CXCL is mainly involved in leukocyte migration, and there is evidence that it plays a role in the pathophysiology of TB (Komolafe and Pacurari, 2022). While its exact function is unknown, CXCL8 was elevated at tuberculin skin test (TST) positive sites in TB sputum samples. On the other hand, blood CXCL8 levels are strongly associated with the severity of TB-induced acute respiratory distress syndrome (ARDS) (Hashemian et al., 2014). CXCL8 is associated with tuberculous granuloma formation and protective immunity against Mtb. Fibroblasts strongly express CXCL8 in the human tuberculous granuloma. A study has shown that the novel C-type lectin receptor CLEC9A mediates activation of the spleen tyrosine kinase (SYK) and mitogen-activated protein kinase (MAPK) pathways after binding to Mtb, selectively up-regulating CXCL8 and neutrophils by chemokines such as interleukin-8 (IL-8/CXCL8) after pathogen invasion (Cheng et al., 2017). After pathogen invasion, neutrophils are activated by chemokines such as interleukin-8 (IL-8/CXCL8), and the conformation of G protein-coupled receptors (GPCRs) is altered, which in turn promotes neutrophil recruitment in the infected area (Manz and Boettcher, 2014). Exogenous CXCL8 can reduce the survival of Mtb in macrophages, and inhibition of CXCL8 can promote the proliferation of intracellular Mycobacterium (Alessandri et al., 2006; O’ Kane et al., 2007). Chemokines such as CXCL1 and CXCL10 were elevated in TB-infected patients and decreased after up to 6 months of anti-TB therapy. Statistical analysis showed that CXCL had a specificity and sensitivity of up to 80% in the diagnosis of active TB and is one of the indicators of disease progression (Zhao et al., 2018; Kumar et al., 2021). Our sequencing results found that CXCL acts as on the signaling pathways of the top DEG sequencings, such as Cytokine-cytokine receptor interactions, the JAK-STAT signaling pathway, the TNF signaling pathway, the IL-17 signaling pathway, the NF-κB signaling pathway, and the C-type lectin receptor signaling pathway. This further confirms the essential role of CXCL in TB infection, suggesting that this molecule may be a highly effective target for novel TB vaccines or drugs.

Mtb is prone to aggregation during growth, and infection with aggregated Mtb leads to early upregulation of pro-inflammatory genes and enhanced TNF-α signaling via the NF-κB pathway. These pathways were significantly up-regulated following the infection of each cell with single or multiple non-aggregating bacilli (Rodel et al., 2021). The TNF signaling pathway is vital to restricting Mtb growth, and interactions between Mtb growing under hypoxia and host macrophages can induce the activation of the TNF signaling pathway, the DNA damage stress response, and apoptosis (Zhang et al., 2020). It has been reported that the JAK-STAT pathway contributes to the expression of multiple pro-inflammatory cytokines to clear Mtb (Zhong et al., 2017; Wu et al., 2022). IL-17 is secreted by Th17 cells, a subset of CD4^+^ T lymphocytes. IL-17A enhances macrophage clearance of intracellular Mycobacterium via a no-dependent killing mechanism and enhances explicitly BCG-induced JAK-STAT pathway phosphorylation in macrophages (Gu et al., 2013; Ling et al., 2013). Pai and Rodrigues (Pai and Rodrigues, 2015) identify the indirect signs of Mtb exposure using the IFN-γ release test, suggesting that there is a cellular immune response to Mtb. Studies have shown that Th17 cell differentiation induces neutrophilic inflammation, mediates tissue damage, and is involved in the pathophysiologic mechanism behind TB (Lyadova and Panteleev, 2015). This cell population plays a protective role in the early stage of TB but leads to disease progression in its late stages. Inhibition of the NF-κB signaling pathway is believed to reduce the inflammatory response to Mtb effectively (Yang et al., 2019), BCG (attenuated *Mycobacterium bovis*) can stimulate host secretion of TNF-α and IL-1β and activate MAPKs/NF-κB signaling pathway to induce the apoptosis of macrophages and the inflammatory response (Yuk et al., 2009; Ma et al., 2014), thereby stimulating pro-inflammatory cytokine production (Li et al., 2020). An increasing number of CTLRs have been studied for their functional role in the pathogenesis of TB (Goyal et al., 2016), and it is believed that they may contribute to the immune escape of Mycobacterium. This study has several limitations, which include its inability to evaluate the specific mechanisms of action of the central genes and related signaling pathways in TB. These require further exploration.

In brief, we identified 597 DEGs whose expression were altered in Mtb-Ag-stimulated PBMCs *vs*. negative controls, of which 345 were up-regulated, and 252 were down-regulated. Significantly impacted pathways included the TNF signaling pathway, the IL-17 signaling pathway, the JAK-STAT signaling pathway, cytokine-cytokine receptor interactions, the NF-κB signaling pathway, and the C-type lectin receptor signaling pathway, which are known to be primarily associated with TB. We screened 10 hub genes by PPI network and validated 34 DEGs using qRT-PCR, with findings consistent with our RNA-seq results. These hub genes can be used as biomarkers for TB diagnosis or prognosis and may provide new directions for the development of potential molecular targets for TB treatment.

## Data availability statement

The data presented in the study are deposited in the https://www.ncbi.nlm.nih.gov/bioproject/PRJNA1017834, accession number PRJNA1017834.

## Ethics statement

The studies involving human participants were reviewed and approved by the Human Ethics Committee of Bengbu Medical College (2022-68). The studies were conducted in accordance with the local legislation and institutional requirements. The participants provided their written informed consent to participate in this study. The study was conducted in accordance with the local legislation and institutional requirements.

## Author contributions

JW: Writing – original draft, Conceptualization, Data curation, Formal Analysis, Investigation, Methodology, Software. FG: Data curation, Formal Analysis, Investigation, Methodology, Writing – original draft. YS: Data curation, Methodology, Writing – original draft. KX: Data curation, Writing – original draft. FL: Data curation, Writing – original draft. KL: Data curation, Writing – original draft. BL: Methodology, Resources, Writing – review & editing. ZQ: Methodology, Writing – review & editing. XW: Methodology, Writing – review & editing. HW: Methodology, Resources, Supervision, Validation, Writing – review & editing. TX: Conceptualization, Funding acquisition, Investigation, Methodology, Resources, Supervision, Writing – review & editing.

## Supplementary material

Supplementary Figure 1 Preparation and identification of Mtb-Ag. **(A)** SDS-PAGE electrophoretic analysis of Mtb-Ag. **(B)** Flow chart of γδ T cells proliferation by Mtb-Ag-stimulated. **(C)** Statistical analysis of γδ T cells proliferation (****P* < 0.001).
