# Supplementary figures and images for: Transcriptional analysis of human peripheral blood mononuclear cells stimulated by Mycobacterium tuberculosis antigen
Source: Front Cell Infect Microbiol. 2023 Sep 25;13:1255905. doi: 10.3389/fcimb.2023.1255905 (PMC10561294; doi:10.3389/fcimb.2023.1255905)

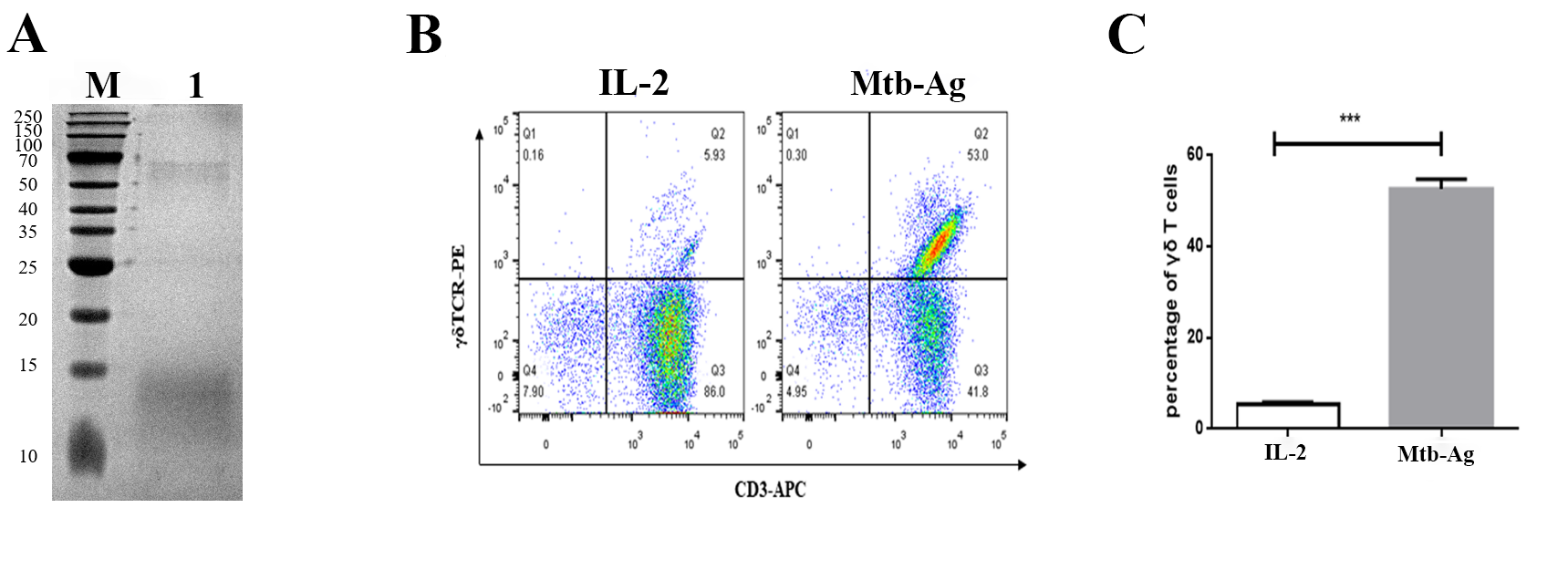

Supplement: Supplementary Figure 1 — Preparation and identification of Mtb-Ag. (A) SDS-PAGE electrophoretic analysis of Mtb-Ag. (B) Flow chart of γδ T cells proliferation by Mtb-Ag-stimulated. (C) Statistical analysis of γδ T cells proliferation (***P < 0.001). [file Image_1.tif]
